# Prediction of pediatric dose of tirzepatide from the reference adult dose using physiologically based pharmacokinetic modelling

**DOI:** 10.3389/fphar.2023.1326373

**Published:** 2023-11-28

**Authors:** Ruifang Guan, Xuening Li, Guo Ma

**Affiliations:** ^1^ Department of Clinical Pharmacology, Zhongshan Hospital, Fudan University, Shanghai, China; ^2^ Department of Clinical Pharmacy, School of Pharmacy, Fudan University, Shanghai, China

**Keywords:** physiologically based pharmacokinetic (PBPK) modelling, tirzepatide, PK-Sim, MoBi, pediatric dose, diabetes

## Abstract

Tirzepatide is an emerging hypoglycemic agent that has been increasing used in adults, yet its pharmacokinetic (PK) behavior and dosing regimen in pediatric population remain unclear. This study aimed to employ the physiologically based pharmacokinetic (PBPK) model to predict changes of tirzepatide exposure in pediatric population and to provide recommendations for its dose adjustments. A PBPK model of tirzepatide in adults was developed and verified by comparing the simulated plasma exposure with the observed data using PK-Sim&MoBi software. This model was then extrapolated to three specific age subgroups, i.e., children (10–12 years), early adolescents (12–15 years), and adolescents (15–18 years). Each subgroup included healthy and obese population, respectively. All known age-related physiological changes were incorporated into the pediatric model. To identify an appropriate dosing regimen that yielded PK parameters which were comparable to those in adults, the PK parameters for each aforementioned subgroup were predicted at pediatric doses corresponding to 87.5%, 75%, 62.5%, and 50% of the adult reference dose. According to the results of simulation, dose adjustments of tirzepatide are necessary for the individuals aged 10–12 years, as well as those aged 12–15 years with healthy body weights. In conclusion, the adult PBPK model of tirzepatide was successfully developed and validated for the first time, and the extrapolated pediatric model could be used to predict pediatric dosing regimen of tirzepatide, which will provide invaluable references for the design of future clinical trials and its rational use in the pediatric population.

## 1 Introduction

The incidence of youth-onset type 1 diabetes mellitus (T1DM) and type 2 diabetes mellitus (T2DM) is increasing, which imposes a growing public health burden (Pihoker et al., 2023). The prevalence of T1DM and T2DM are projected to be 5.2 cases and 0.8 cases per 1000 youth, respectively (Dabelea et al., 2021). Obesity is believed to be one of the major modifiable risk factors for T2DM (Zhou et al., 2023). Studies indicated that obesity in childhood and adolescence increases the risk of youth-onset T2DM (Malone and Hansen, 2019). Current pharmacologic treatment options for children and adolescents with diabetes are limited to insulin, metformin, and two glucagon-like peptide-1 (GLP-1) receptor agonists, i.e., daily liraglutide and once-weekly exenatide extended release (ElSayed et al., 2023).

Glucose-dependent insulinotropic polypeptide (GIP) and GLP-1, as two main incretin hormones, are responsible for glucose homeostasis and glucose-dependent insulin secretion (Hammoud and Drucker, 2023). GLP-1 and GIP may improve β-cells functionality through synergistical pharmacological activation (Bastin and Andreelli, 2019). Tirzepatide is the first dual GIP and GLP-1 receptor co-agonist, approved by the Food and Drug Administration (FDA) and the European Medicine Agency (EMA) recently. Comparing to other drug treatments, once-weekly tirzepatide had shown superior glycemic and body weight control capacity with a comparable safety profile (Guan et al., 2022; Shi et al., 2023). Moreover, a cardiovascular risk assessment also favored tirzepatide based on the completed clinical trials (Sattar et al., 2022). Tirzepatide is administered subcutaneous once weekly with a recommended dose-escalation regimen, initiated by 2.5 mg for 4 weeks and increased by 2.5 mg every 4 weeks until reaching a maintenance dose between 5 and 15 mg (Gasbjerg et al., 2023). Although the pharmacokinetics (PK) of tirzepatide have been characterized in adults, no PK or dose-adjustment data of pediatric population have been released up to now. The demand for antidiabetic and weight-reducing medications among the pediatric population is increasing.

Administration of an appropriate dose is critical to obtain optimum systemic drug concentration. Yet unlike the adult population, the recruitment of pediatric patients for clinical investigation is confounded by inherent logistical and ethical constraints (Yellepeddi et al., 2019). Thus, the pediatric dose is usually calculated from the adult dose using empirical formulae based on the age, body weight and body surface area (Rashid et al., 2020). These methods ignore all the age-dependent physiological differences which may affect the PK of a drug (Barrett et al., 2012), e.g., blood flow, body composition, ontogeny of metabolic enzymes, and glomerular filtration rate (GFR). In addition, dosing for obese children must take the effect of obesity on PK into account because altered body size may affect drug disposition (Hanley et al., 2010). Physiologically based pharmacokinetic (PBPK) modelling is recommended by the FDA for pharmaceutical research and dose selection in pediatric patients (Khalil and Läer, 2014), since it’s a more advanced and comprehensive approach to predict PK parameters by integrating known physiological changes that may alter drug absorption and disposition in pediatric population (van der Heijden et al., 2023). Currently, PBPK modelling is an increasingly-popular and extensively-used strategy to extrapolate a drug’s PK from adults to children, and to support dosing decisions (Yellepeddi et al., 2019).

The purpose of this study is to develop and validate a PBPK model of tirzepatide in adults, extrapolate it to children and adolescents with both healthy and obese body weights, and simulate the exposure of tirzepatide in the pediatric population. An appropriate dosing regimen for pediatric patients can be proposed by comparing and matching pediatric drug exposures with that in adults. This unprecedented PBPK model can fulfill the gaps in the knowledge of the rational use of tirzepatide in children and adolescents, and provide references for designing clinical trials in future.

## 2 Methods

### 2.1 Software

A PBPK model of tirzepatide was developed and verified in the adult population using PK-Sim and MoBi, which are part of the Open Systems Pharmacology Suite 11.0. This software suite (released under the GPLv2 license by the Open Systems Pharmacology community, www.open-systems-pharmacology.org) is user-friendly, open-source, and accredited for PBPK modelling. The model was then scaled to the pediatric population. The plasma concentration-time curve data of tirzepatide were extracted with the GetData Graph Digitizer 2.24 according to best practices (Wojtyniak et al., 2020). Pharmacokinetic parameters analysis, statistical calculations, and plots generation were performed in R 4.1.1 (R Foundation for Statistical Computing, Vienna, Austria).

### 2.2 Model development and verification in adults

**FIGURE 1:**
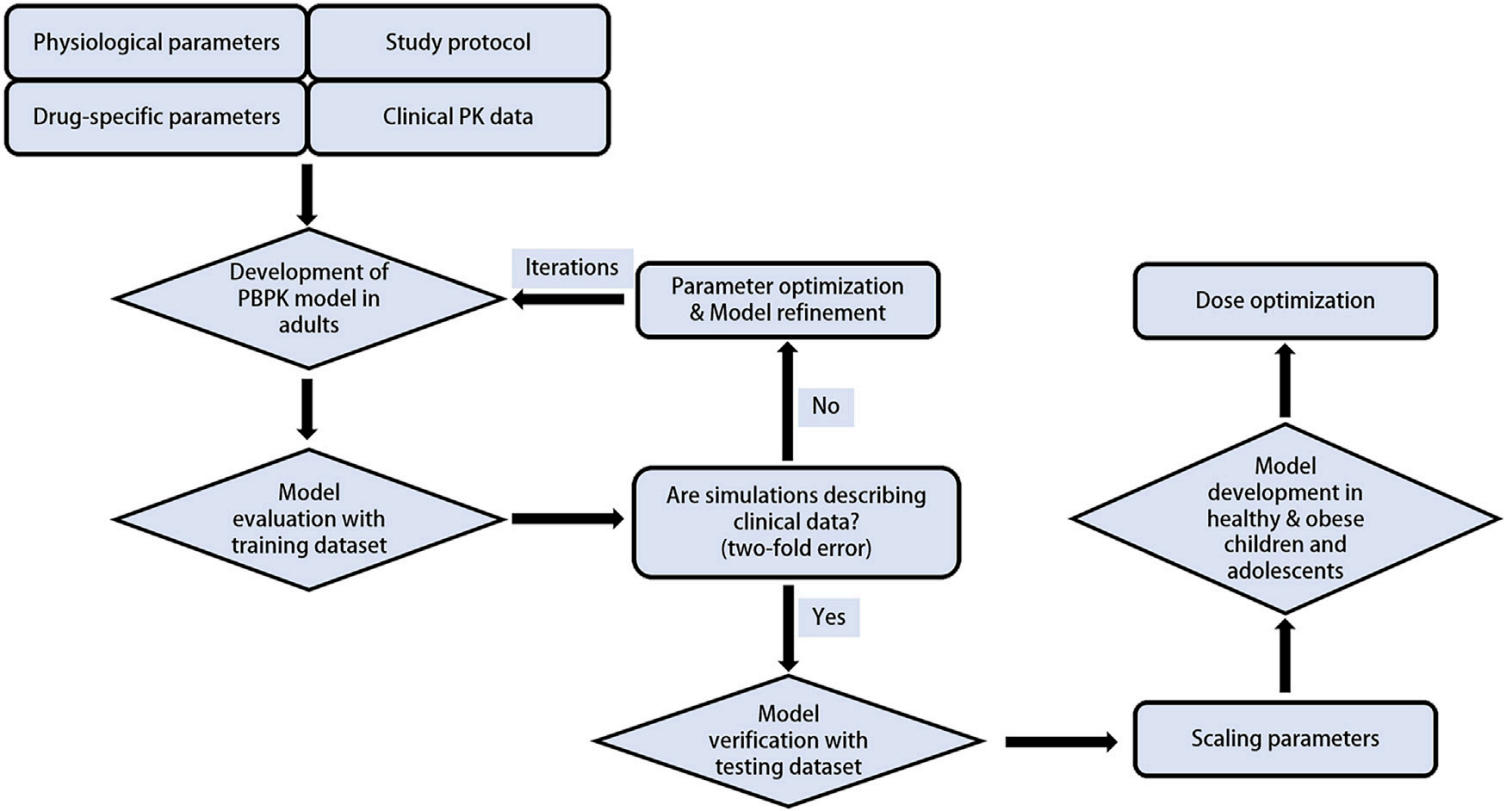
Schematic representation of the PBPK modeling workflow.

The PBPK modelling was performed in a stepwise procedure as shown in Figure 1. Drug-specific physicochemical properties, as well as information on the absorption, distribution, metabolism, and elimination characteristics were obtained from comprehensive literature search or parameter optimization. A total of 5 clinical trials including PK data of tirzepatide were gathered and used to develop and validate the PBPK model. Collected plasma concentration-time profiles were split into a training dataset for model development and parameter optimization, and a testing dataset for model evaluation.

A “middle-out” strategy, which integrates both “bottom-up” and “top-down” approaches, was utilized. In this method, key parameter estimates (e.g., CL_kidney_) were optimized using the built-in Monte-Carlo algorithm in MoBi. Subcutaneous dosing was modeled by optimizing a first-order absorption process described with Eq. 1 (Dubbelboer and Sjögren, 2022) using data from the training dataset of adults receiving 5 mg of tirzepatide (Coskun et al., 2018).

$$\frac{dAsc}{dt}=-Ka\times Asc;Asc_{0}=F\times \text{Dose}$$ (1)

*A*_*SC*_ describes the drug amount in the SC administration site, *Ka* the absorption rate, *F* the bioavailability, and *Dose* the administered dose.

Tirzepatide is extensively metabolized through a process similar to proteolysis, therefore we customized a hypothetical peptidase that was defined to be widely expressed throughout the body at a concentration of 1 μmol/L in organs and tissues. Tirzepatide with a relatively low molecule weight (<5 kDa) is assumed to be filtered freely by glomerulus (Meibohm, 2007) and metabolized in the proximal tubule to some extent, where many enzymes including peptidases are located (Brater, 2002). The model accounted for extensive systemic metabolism through proteolytic cleavage via peptidases and renal elimination through kidney clearance, with the fraction of the drug eliminated in the urine fixed at 66%, as reported in the FDA Clinical Pharmacology Review (FDA, 2021).

The developed tirzepatide PBPK model was first evaluated by visually comparing simulated vs. observed concentration–time profiles, using single and multiple dosing regimens of clinical data from the testing dataset (Urva et al., 2021; Furihata et al., 2022; Urva et al., 2022; Feng et al., 2023). Additionally, primary PK parameters, i.e., the maximum concentration (C_max_), area under the curve from 0 to infinity time (AUC_0-inf_), half-life (T_1/2_), the time to reach peak concentration (T_max_), and clearance over bioavailability (CL/F), were compared between predictions and observations using the fold error and the average fold error (AFE), calculated according to Eq. 2 and Eq. 3, respectively. The PBPK model is considered acceptable if fold error and AFE are within the 0.5–2 range (Kuepfer et al., 2016).

$$\text{fold error}=\frac{Observed\;PK\;parameter}{Predicted\;PK\;parameter}$$ (2)

$$\text{AFE}=10^{\frac{\sum \log\;\left(fold\;error\right)}{N}}$$ (3)

### 2.3 Model extrapolation to children and adolescents

The tirzepatide PBPK model developed and verified in adults was then scaled to children and adolescents (10–18 years) with both healthy and obese body weights. According to previously established guidelines for scaling PBPK models from adults to pediatric population, model structure and clearance processes were considered similar between these two groups (Maharaj and Edginton, 2014). Therefore, in the pediatric model, drug-specific inputs were maintained unaltered, and age-related physiological parameters including height, weight, organ volume, cardiac output and blood flow rate were modified based on the built-in algorithm of PK-Sim (Edginton et al., 2006b). No age-related maturation of drug-metabolizing enzymes or changes in the unbound fraction were considered in this study, since the PBPK models were scaled to individuals aged 10 years and above. For this age, the maturity level of enzymes and the abundance of albumin are considered to be no different from those of adults (McNamara and Alcorn, 2002). Besides, CL_renal, pediatric_ (renal clearance of the pediatric population) was calculated as shown in Eq. 4 (Edginton et al., 2006a).

$$CL_{renal,pediatric}=\frac{{GFR}_{pediatric}}{{GFR}_{adult}}*\frac{{fu}_{pediatric}}{{fu}_{adult}}*CL_{renal,adult}$$ (4)

*GFR*_*pediatric*_ and *GFR*_*adult*_ are the GFR in pediatrics and adults, *fu* is the unbound fraction, *CL*_*renal, adult*_ is the optimized renal clearance in the adult model.

The pediatric population was grouped based on age into three subgroups, i.e., children (10–12 years), early adolescents (12–15 years) and adolescents (15–18 years). Each virtual pediatric population consisted of 50 females and 50 males. For those with normal body weights, simulations were performed using the default virtual individuals and populations embedded in the PK-Sim (Ford et al., 2022). For those with obese body weights, the validated virtual population (Gerhart et al., 2022b) accounted for key obesity-related physiological changes relevant to PK (i.e., body weight, body composition, organ size, blood flow and glomerular filtration rate (GFR)), was adopted. The characteristics of the virtual pediatric population used for the model development were shown in Supplementary Table S1 of the Electronic supplementary material (ESM). Briefly, body weights were increased to reflect a body mass index (BMI) greater than the 95th percentile (Gulati et al., 2012) as defined by the growth charts from the US Centers for Disease Control and Prevention. Body weights were also redistributed between adipose tissue and lean organs, with an increase of 115% in kidney and liver. Other organ volume scaling factors were shown in Supplementary Table S2 of the ESM. Followed with increased organ volumes, cardiac output and organ blood flow as well as absolute GFR, were also elevated.

Since 5 mg is the minimum maintenance dose of tirzepatide in adults, it’s chosen as the adult reference dose in this study. The simulated concentration-time profile and primary PK parameters in pediatric population were compared with observed data in adults. Then, the PK parameters were recalculated by decreasing the doses from 5 mg to 4.375, 3.75, 3.125, and 2.5 mg, which corresponded to dose reduction of 12.5%, 25%, 37.5%, and 50% respectively to accomplish the desired PK parameters within the reference adult ranges.

## 3 Results

### 3.1 Development and verification of PBPK model in adults

**FIGURE 2:**
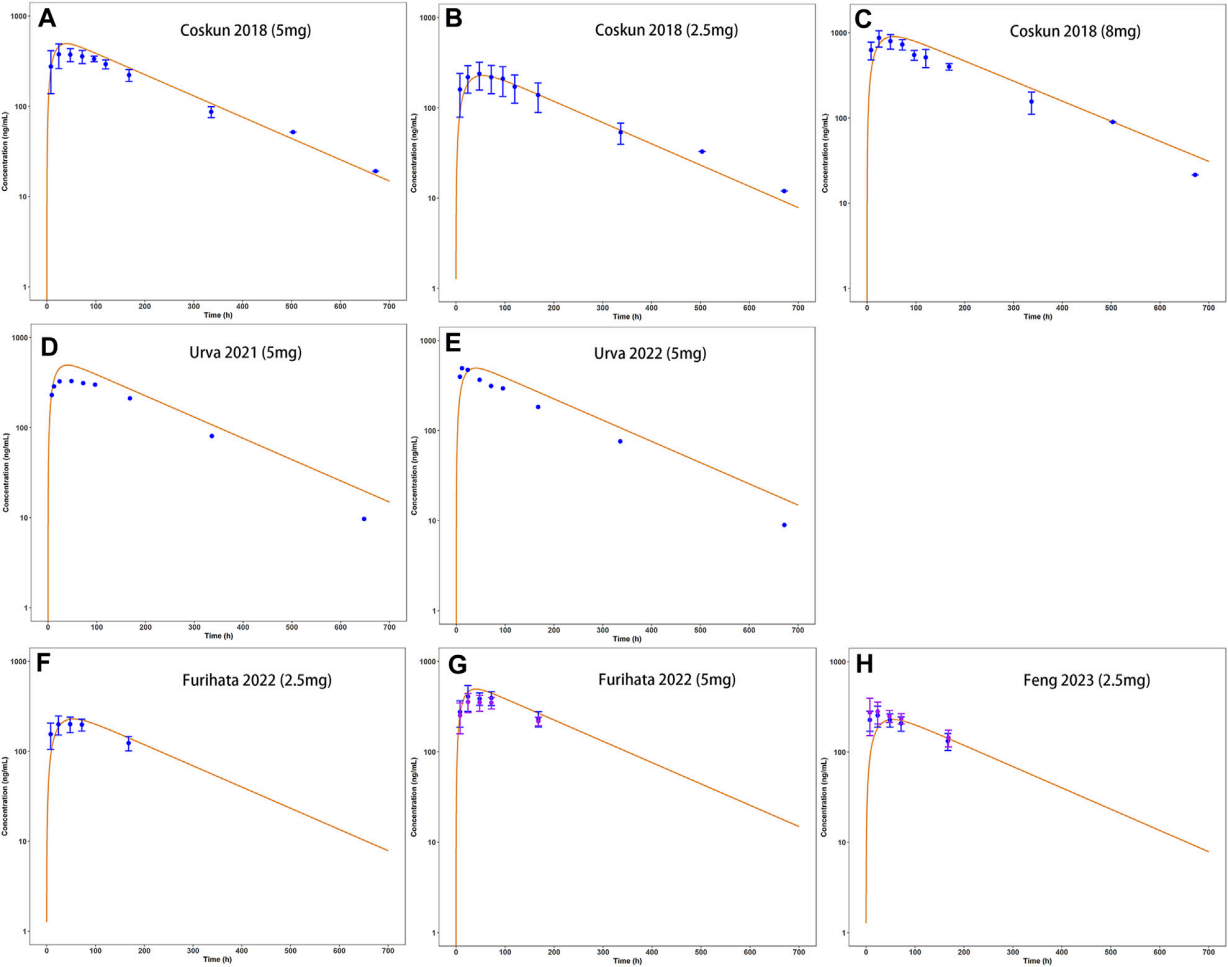
Development **(A)** and external verification **(B**–**H)** of tirzepatide PBPK model in adults after single SC administration (2.5–8 mg). Solid lines indicated simulated arithmetic mean plasma concentration–time profiles. Observed data was shown as circles ±standard deviation if available.

**FIGURE 3:**
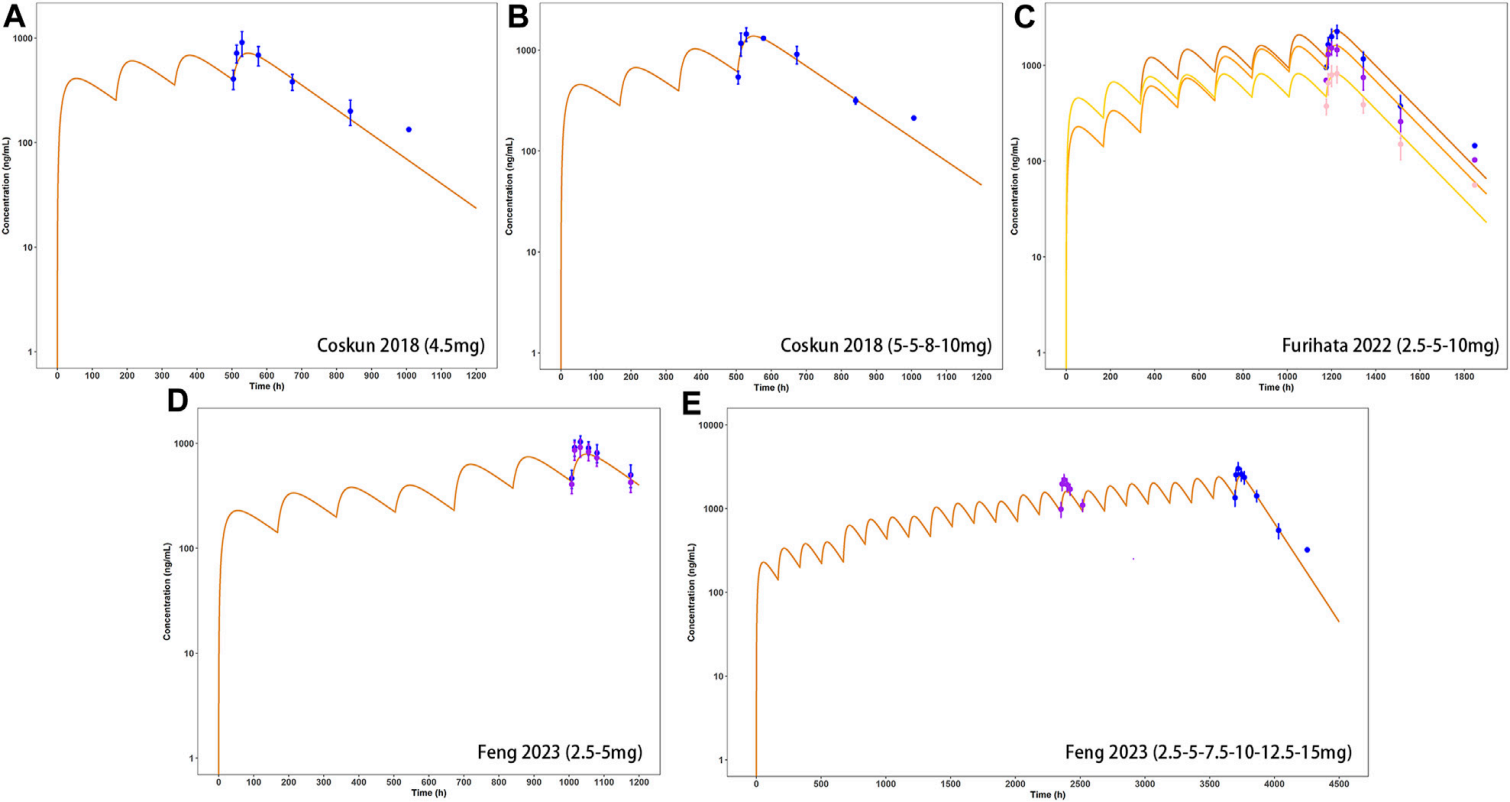
External verification (A–E) of tirzepatide PBPK model in adults after multiple dosing regimens (details can be found in Table 4). Solid lines indicated simulated arithmetic mean plasma concentration–time profiles. Observed data was shown as circles ±standard deviation if available.

Observed plasma drug concentration data of adults receiving 5 mg single dose of tirzepatide in a clinical trial (Coskun et al., 2018) was chosen as training dataset for parameter optimization and the development of the adult model (Figure 2A), data from 5 clinical studies covering a broad range of 0.25–15 mg in single (Figures 2B–H) and multiple (Figures 3A–E) dosing regimens were used as testing dataset for model external verification. The details of the included studies were summarized in Table 1. Drug-related input parameters for constructing the PBPK model were listed in Table 2.

**TABLE 1:** Description of population characteristics of included clinical studies for model development and validation.

Characteristics	Training dataset	Testing dataset			
	Coskun et al. (2018)	Furihata et al. (2022)	Urva et al. (2021)	Urva et al. (2022)	Feng et al. (2023)
Number of female/male subjects	3/53	1/47	5/9	3/10	11/13
Population	Asian	Japanese	European	European	Chinese
Dosage regimen (mg)	single/multiple dose 2.5/5/8	single/multiple dose 2.5/5/10/15	single dose 5	single dose 5	single/multiple dose 2.5/5/10/15
Age (years)	39.4 ± 10.3	57.4 ± 8.8	58.1 ± 7.6	55.8 ± 11.3	56.3 ± 5.4
Weight (kg)	71.9 ± 11.1	72.3 ± 10.4	88.0 ± 11.2	96.72 ± 18.62	67.1 ± 7.6
BMI (kg/m^2^)	24.7 ± 3.2	25.4 ± 3.2	28.6 ± 2.6	30.88 ± 4.57	25.7 ± 2.3

**TABLE 2:** Parameters used in tirzepatide PBPK model development.

Parameters	Value	Reference
Physicochemical properties		
Molecular Weight (g/mol)	4813.5	FDA label
Log P	−6.8	Pubchem
Solubility	<1 mg/mL	Drug Bank
fu	1%	Drug Bank
**Absorption**		
Ka (1/h)	0.0996	Optimized
Bioavailability	81%	FDA label
**Distribution**		
Partition coefficients	PK-Sim Standard	Willmann et al. (2005)
Cellular permeabilities	PK-Sim Standard	Willmann et al. (2005)
**Metabolism**		
peptidase		
CL_spec_ (l/μmol/min)	0.35	Optimized
**Excretion**		
GFR fraction	1.00	FDA label
f_urine_	66%	FDA label
Renal clearance (1/min)	0.12	Optimized

Log P, logarithm of octanol/water partition coefficient; fu, fraction unbound in plasma; CL_spec_, specific clearance by peptidase in PK-Sim; FDA, US, food and drug administration; f_urine_, fraction excreted to urine; Ka, absorption rate constant; GFR, glomerular filtration rate. A GFR, fraction of 1.0 in PK-Sim indicates no tubular secretion or reabsorption is included.

Model-predicted concentration-time profiles are in close concordance with the corresponding observed data as shown in Figure 2; Figure 3. Table 3 and Table 4 displayed the predicted and observed primary PK parameters, as well as their fold error for single and multiple dosing regimens, respectively. The majority of the fold errors fell in the range of 0.5–2, except for T_max_ (0.83–2.28 h). This was expected due to the high interindividual variability of T_max_, and the simulated values were all within the observed range of clinical studies (24–72 h) (FDA, 2021). Additionally, the AFE of single dose regimen was computed with values of 1.18, 1.09, 1.04, 1.42, and 0.84 for AUC_0-inf_, C_max_, T_1/2_, T_max_ and CL/F, respectively. All the values fell in the 2-fold range, which further validated the developed model.

**TABLE 3:** Comparison of PBPK model simulated and observed PK parameters following single-dose SC administration in adults.

Parameters (Units)	Dose (mg)		AUC_0-inf_ (ng·h/mL)	C_max_ (ng/mL)	T_1/2_ (h)	T_max_ (h)	Cl/F (L/h)
Coskun et al. (2018)	2.5	Predicted	56546	228.1	128.3	54.7	0.0440
		Observed	53200	231.0	120.0	24.0	0.0470
		**Fold error**	**1.06**	**0.99**	**1.07**	**2.28**	**0.94**
	5	Predicted	113085	494.2	128.0	40.0	0.0442
		Observed	90500	397.0	123.0	24.1	0.0553
		**Fold error**	**1.25**	**1.24**	**1.04**	**1.66**	**0.80**
	8	Predicted	223391	901.4	128.3	54.7	0.0358
		Observed	169000	874.0	111.0	48.0	0.0472
		**Fold error**	**1.32**	**1.03**	**1.16**	**1.14**	**0.76**
Furihata et al. (2022)	5	Predicted*	113085	494.2	128.0	40.0	NA
		Observed	104000	364.0	127.0	48.0	NA
		**Fold error**	**1.09**	**1.36**	**1.01**	**0.83**	**1.54**
Urva et al. (2021)	5	Predicted	113084	494.2	128.0	40.0	0.0442
		Observed	80500	339.0	121.0	48.0	0.0621
		**Fold error**	**1.40**	**1.46**	**1.06**	**0.83**	**0.71**
Urva et al. (2022)	5	Predicted	113084	494.2	128.0	40.0	0.0442
		Observed	84300	510.0	124.0	24.0	0.0593
		**Fold error**	**1.34**	**0.97**	**1.03**	**1.67**	**0.75**
Feng et al. (2023)	2.5	Predicted*	30673	228.8	128.3	54.7	0.0441
		Observed	35100	306.0	139.0	24.0	0.0384
		**Fold error**	**0.87**	**0.75**	**0.92**	**2.28**	**1.15**

AUC_0-inf_, area under the plasma concentration–time curve from time zero to infinity; C_max_, maximum plasma concentration; t_1/2_, half-life; t_max_, time to reach peak concentration; CL/F, clearance over bioavailability; NA, not applicable. *AUC, is calculated between 0 and 168 h. Fold error was calculated according to Eq. 2.

**TABLE 4:** Comparison of PBPK model simulated and observed PK parameters following multiple-dose SC administration in adults.

Parameters (Units)	Dose (mg)	Dosing regimen		AUC_0–168h_ (ng·h/mL)	C_max_ (ng/mL)	T_1/2_ (h)	T_max_ (h)
Coskun et al. (2018)	4.5	4.5 mg (4)*	Predicted	988780	717.5	128.3	42.0
			Observed	103000	884.0	132.0	24.2
			**Fold error**	**0.96**	**0.81**	**0.97**	**1.74**
	5-5-8-10	5 mg (2), 8 mg (1), 10 (1)	Predicted	189535	1378.0	128.3	45.3
			Observed	198000	1510.0	126.0	24.2
			**Fold error**	**0.96**	**0.91**	**1.02**	**1.87**
Furihata et al. (2022)	5	5 mg (8)	Predicted	112988	819.8	128.3	41.2
			Observed	104000	838.0	127.0	48.0
			**Fold error**	**0.92**	**1.02**	**0.99**	**1.17**
	10	2.5 mg (2), 5 mg (2), 10 mg (4)	Predicted	222479	1614.2	128.3	41.0
			Observed	192000	1520.0	135.0	24.0
			**Fold error**	**1.16**	**1.06**	**0.95**	**1.71**
	15	5 mg (2), 10 mg (4), 15 mg (2)	Predicted	318900	2314.5	128.3	42.7
			Observed	285000	2270.0	121.0	48.0
			**Fold error**	**1.12**	**1.02**	**1.06**	**0.89**
Feng et al. (2023)	5	2.5 mg (4), 5 mg (4)	Predicted	108794	789.5	130.6	42.0
			Observed	110000	915.0	124.0	24.0
			**Fold error**	**0.99**	**0.86**	**1.05**	**1.75**
	10	2.5 mg (4), 5 mg (4), 7.5 mg (4), 10 mg (4)	Predicted	221867	1609.9	128.4	41.8
			Observed	263000	2200.0	132.0	24.0
			**Fold error**	**0.84**	**0.73**	**0.97**	**1.74**
	15	2.5 mg (4), 5 mg (4), 7.5 mg (4), 10 mg (4), 12.5 mg (4), 15 mg (4)	Predicted	336231.5	2439.7	128.25	41.5
			Observed	357000	2930	126	24
			**Fold error**	**0.94**	**0.83**	**1.02**	**1.73**

AUC_0–168h_, area under the plasma concentration–time curve during one dose interval after last dose; C_max_, maximum plasma concentration; T_1/2_, half-life; T_max_, time to reach peak concentration; *The number in parentheses represented how many weeks the dose was used. Fold error was calculated according to Eq. 2.

### 3.2 Simulated pharmacokinetic profiles in children and adolescents at adult dose

**FIGURE 4:**
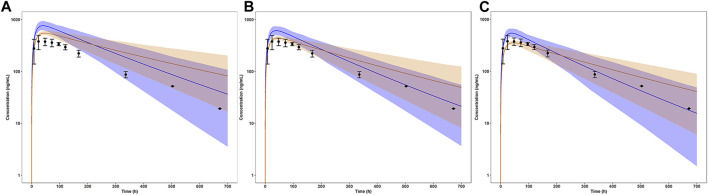
Simulated mean concentration-time profiles in children and adolescents with normal and obese body weights after a single dose of 5 mg. **(A)** Children (10–12 years); **(B)** Early Adolescents (12–15 years); **(C)** Adolescents (15–18 years). The blue line represented the normal weight group, the orange line represented the obese weight group. The light shaded regions represented the 90% model prediction interval. Observed data in adults was shown as black circles ±standard deviation if available.

**FIGURE 5:**
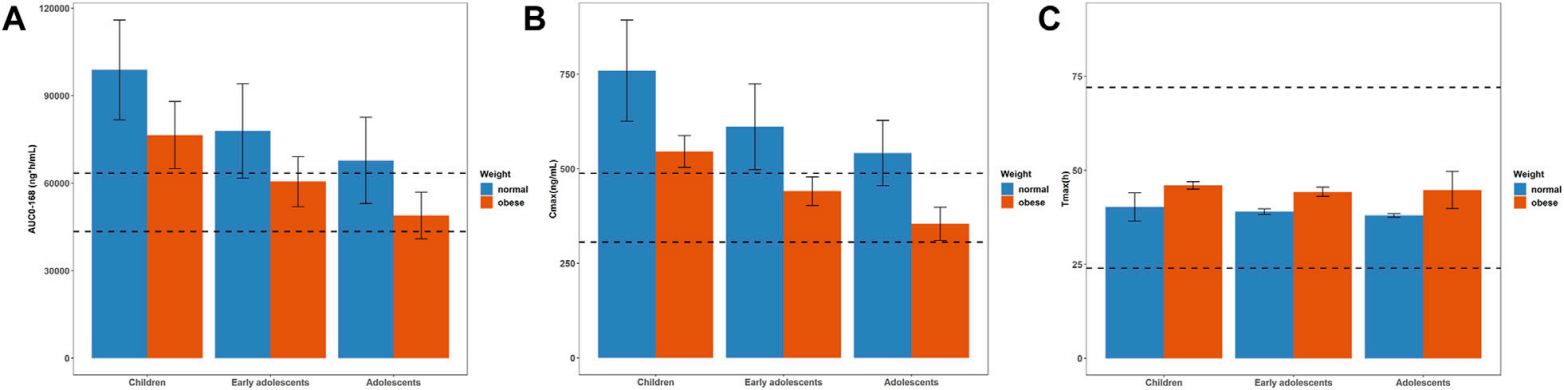
Simulated mean PK parameters and standard deviation in children (10–12 years), early adolescents (12–15 years) and adolescents (15–18 years) with normal and obese body weights after a single dose of 5 mg. **(A)** AUC_0–168h_; **(B)** C_max_; **(C)** T_max_. The range of PK parameters of adults receiving a single dose of 5 mg, such as 43459–63467 ng*h/mL for AUC_0–168h_, 305.7–488.3 ng/mL for C_max_, and 24–72 h for T_max_, were used as the reference range, represented as black dotted lines.

The simulation results of pediatric population receiving 5 mg single dose with comparison to the reference adult values were shown in Figure 4 (concentration-time profile) and Figure 5 (PK parameters). As for healthy and obese children (10–12 years), the lower limits of predicted AUC_0–168h_ and C_max_ both exceeded the reference range. It’s a similar story for the early adolescents (12–15 years) with normal body weights, yet for those with obese body weights, the simulated PK parameters fell in the reference range. In terms of both healthy and obese adolescents (15–18 years), the simulated AUC_0–168h_ and C_max_ were both overlapped with the reference range. T_max_ of each group fell in the reference range.

### 3.3 Pediatric dose simulations

**FIGURE 6:**
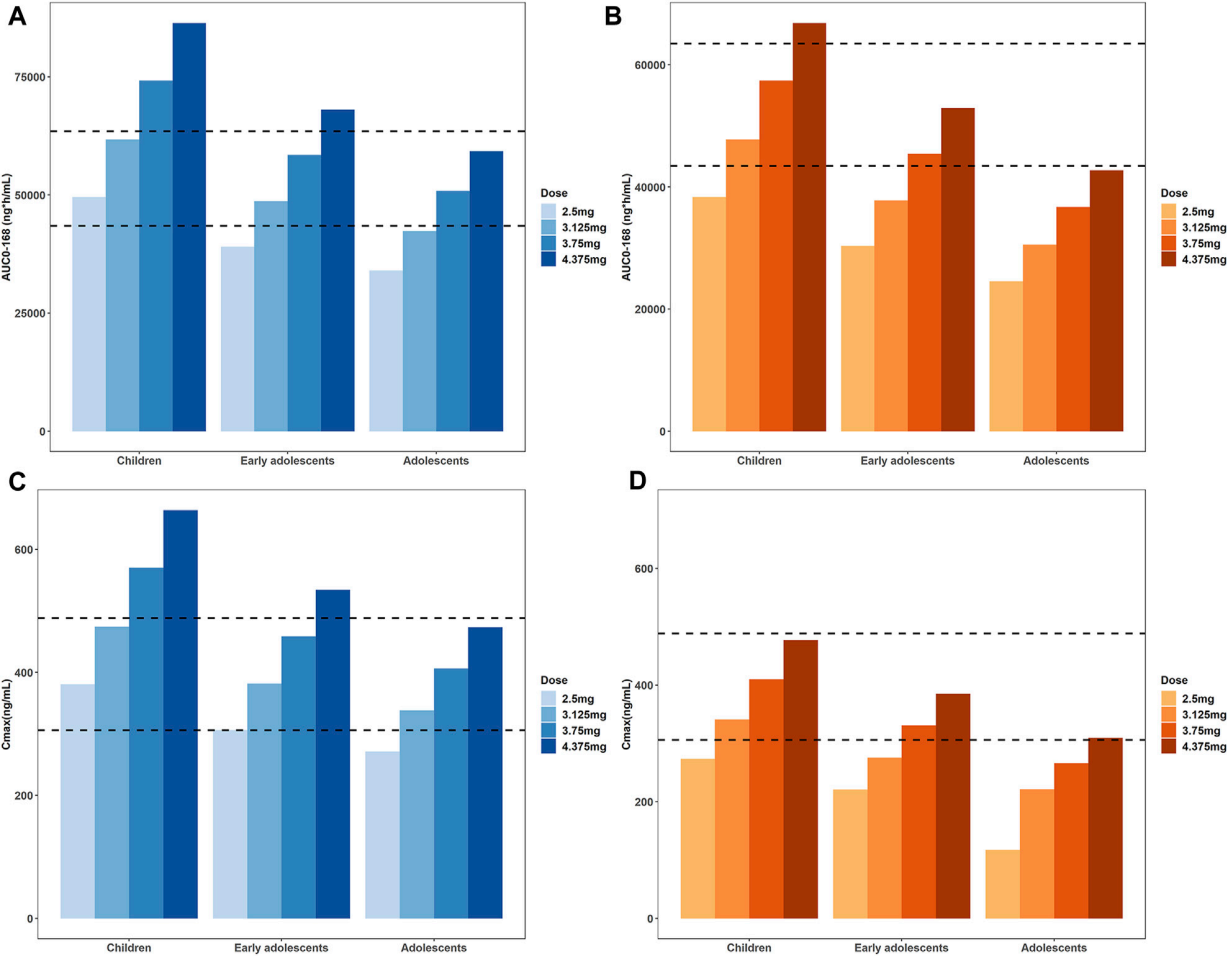
Simulated AUC_0–168h_ **(A,B)** and C_max_ **(C,D)** at different single doses to obtain values in healthy and obese pediatric population similar to the reference adult range, i.e. 43459- 63467 ng*h/mL for AUC_0–168h_ and 305.7–488.3 ng/mL for C_max_. Those with normal body weights were represented in the blue bar charts **(A)** and **(C)**, the obese population was represented in the orange bar charts **(B)** and **(D)**.

As expected, the predicted plasma concentration and PK parameters of tirzepatide in pediatric population at the adult dose (5 mg) were higher, indicating dose adjustments were needed. Hence, dose of tirzepatide is reduced to 87.5%, 75%, 62.5%, and 50% of the reference adult dose to obtain both desirable AUC_0–168h_ and C_max_. The simulated PK parameters at different single doses were shown in Figure 6 and Supplementary Table S3 of the ESM. Appropriate dose adjustments predicted by PBPK modelling were presented in Table 5. For children with normal body weights, the preferred dosage was 2.5–3.125 mg, which corresponded to 50%–62.5% of the adult dose. For obese children and healthy early adolescents, the recommended dosage was 3.125–3.75 mg, corresponding to 62.5%–75% of the adult dose. For obese early adolescents and healthy adolescents, 3.75–5 mg of tirzepatide was recommended, which was equivalent to 75%–100% of the adult dose. No dosage adjustment was necessary for adolescents (15–18 years) with obese body weights according to the simulation results. In addition, predicted concentration-time profiles at each dose in normal and obese pediatric population were demonstrated in Supplementary Figures S1, S2 of the ESM, respectively.

**TABLE 5:** Dose adjustment for pediatric population with different ages and body weights with PBPK modelling.

Age	Weight	Predicted dose
Children	normal	2.5–3.125 mg
	obese	3.125–3.75 mg
Early adolescents	normal	3.125–3.75 mg
	obese	3.75–5 mg
Adolescents	normal	3.75–5 mg
	obese	---

---No dose adjustment is needed in obese adolescents.

## 4 Discussion

At present, the therapeutic options available for pediatric patients with diabetes are somewhat limited, only insulin, metformin, daily liraglutide and once-weekly exenatide extended release are applied in clinical practice. Tirzepatide demonstrated superior glycemic control and body weight reduction outcomes in clinical trials compared to existing medications. It is anticipated that, if tirzepatide can be adapted for use in the pediatric population, it would yield favorable outcomes and substantially alleviate the burden of diabetes. In this study, we developed a PBPK model of tirzepatide using both *in vitro* and *in vivo* data, and verified the model in adults, then scaled it to children and adolescents with both normal and obese body weights. Appropriate pediatric doses were predicted to achieve similar drug exposures to that in adults. To our knowledge, this is the first established PBPK model for dose prediction of tirzepatide in the pediatric population.

Previously, pediatric doses were usually extrapolated from adults using simple allometric scaling based on age, body weight or body surface area. However, the pediatric population undergoes physiological changes in body weight, body composition, and drug elimination pathways, all of which have profound effects on PK of drugs. Therefore, the use of PBPK modelling to extrapolate the initial doses for pediatric clinical trials has been steadily increasing in the last few years, and recognized by authorities (Heimbach et al., 2019; Johnson et al., 2021). Moreover, it’s estimated more than half of all drugs are prescribed off-label to children. PBPK modelling is a valuable approach to support dosing decisions in the pediatric population (Freriksen et al., 2023). At present, PBPK modelling is widely applied in pre-market studies to guide first-in-pediatric dose selection (Wang et al., 2021), as well as in post-marketing phase to establish pediatric drug dosing recommendations.

Since the prevalence of T2DM is very low in children aged <10 years (0.01 cases per 1,000 individuals) and much higher in older children (0.42 cases per 1,000 individuals) (Liese et al., 2006), the extrapolated age range is limited between 10 and 18 years in our model. Within this age group, the proteolytic enzymes (peptidases) mediating tirzepatide metabolism were considered to achieve similar maturity to that in adults (Chang et al., 2021). Therefore, the same non-renal clearance was used in our model between children and adults. Currently, quantitative information about the absorption and metabolic processes of peptides at the SC injection sites is not available (Kagan, 2014), thus a first-order absorption process between SC injection site to the compartment of venous blood was applied in this model. One previous study showed that the processes governing drug absorption were most likely to be consistent between adults and children (Malik and Edginton, 2018). Proteolysis at the SC tissue is a major elimination pathway for therapeutic protein and peptides that reduce their systemic bioavailability (Varkhede et al., 2020). Studies showed there may be a faster absorption rate and greater extent of pre-systemic elimination of drugs via SC administration in the pediatric population, but overall their bioavailability was the same as that in adults (Malik and Edginton, 2018).

The rising prevalence of obesity confronts physicians and pharmacists with dosing problems in this population. According to a systematic review of clinical studies conducted in obese children, clinically significant PK alterations were found in 65% of drugs studied, including changes in the volume of distribution and clearance (Harskamp-van Ginkel et al., 2015). Physiological alterations associated with obesity, e.g., increased tissue volume and perfusion, altered plasma protein concentrations and tissue composition, may significantly impact the distribution volume of a drug. Consequently, adjustments of initial doses might be necessary. Obesity-related changes in the drug eliminating organs, e.g., variations in hepatic enzyme activity and GFR, potentially warrant adjustments to the maintenance dosages (Gerhart et al., 2022a). Therefore, when prescribing medication for obese patients, it is necessary to consider whether dosage adjustments are required. There is usually a disproportionately low number of obese participants in clinical trials, resulting in insufficient dosing recommendations for this population (Berton et al., 2023). PBPK modelling facilitates the creation of simulated clinical trial scenarios based on prior knowledge on human physiology, permitting the investigation on how drugs are distributed and eliminated in the obese subjects. In the present model, the virtual population of children and adolescents with obesity was developed to reflect a greater total body weight and lean body mass (organ volumes) with obesity compared to those with normal body weights. Higher absolute blood flow in organs, GFR and renal clearances were also taken into consideration.

An adult PBPK model for tirzepatide was successfully developed, and scaled it to the pediatric population incorporating all known physiological changes. Some limitations should be noted. As a result of limited clinical studies, there has been no consensus whether the drug absorption rate or bioavailability from SC injections is altered in obese population so far. A delayed absorption of insulin lispro in obese patients was found (Gagnon-Auger et al., 2010), yet no difference of bioavailability of enoxaparin was observed between obese and nonobese individuals in a study (Sanderink et al., 2002). The AUC of human chorionic gonadotropin was substantially lower in the obese women group (Chan et al., 2003), yet it was challenging to distinguish whether it resulted from decreased absorption or increased clearance. Therefore, the absorption rate and extent are considered as consistent between healthy and obese population in this model. Since information on exposure-response of tirzepatide in children was absent, the relationship was assumed to be similar to that in adults. Besides, the aim of this study was to predict appropriate dosing regimen in children and provide references for future clinical trials, the extrapolated pediatric model was not verified because no PK data has been released up to now. However, the finding of this study could be translatable clinically only after a clinical study was conducted in such patients with the predicted doses.

In summary, a PBPK model of tirzepatide in adults was developed and verified to mechanistically describe the *in vivo* process of tirzepatide after SC administration. Then the model was extrapolated to children and adolescents (10–18 years) to account for age-related physiological changes. The developed pediatric PBPK model can provide invaluable references in designing dosing regimens of tirzepatide for the pediatric population, and contribute to individualized medication as well as rational use of tirzepatide in clinical practice.

## Data Availability

The original contributions presented in the study are included in the article/Supplementary Material, further inquiries can be directed to the corresponding authors.

## Abbreviations

AFE, average fold error; AUC, area under the curve; BMI, body mass index; C_max_, maximum concentration; CL/F, clearance over bioavailability; ESM, electronic supplementary material; F, bioavailability; FDA, Food and Drug Administration; fu, fraction unbound in plasma; GFR, glomerular filtration rate; GIP, glucose-dependent insulinotropic polypeptide; GLP-1, glucagon-like peptide-1; Ka, absorption rate; log P, logarithm of octanol/water partition coefficient; PBPK, physiologically based pharmacokinetic; PK, pharmacokinetic; SC, subcutaneous; T_1/2_, half-life; T_max_, the time to reach peak concentration; T1DM, type 1 diabetes mellitus; T2DM, type 2 diabetes mellitus.
