# Supplementary material for: Prediction of pediatric dose of tirzepatide from the reference adult dose using physiologically based pharmacokinetic modelling
Source: Front Pharmacol. 2023 Nov 28;14:1326373. doi: 10.3389/fphar.2023.1326373 (PMC10714946; doi:10.3389/fphar.2023.1326373)
Supplement: Supplementary file 1 [file DataSheet1.docx]

**Table S1. Characteristics of the virtual population used for model development in adults and adolescents.**

| **Population** | **Size** | **Age** | **Gender** | **Weight** | **Weight**  **(kg)** | **BMI**  **(kg/m^2^)** |
| --- | --- | --- | --- | --- | --- | --- |
| Children | 100 | 10-12 | male | healthy | 32-41.6 | 16.8-18.95 |
|  |  |  |  | obese | 53.7-87.9 | 28.2-39.3 |
|  |  |  | female | healthy | 32-41 | 16.8-18.55 |
|  |  |  |  | obese | 54.3-88.2 | 28.5-39.9 |
| Early adolescents |  | 12-15 | male | healthy | 46.4-51.2 | 19.21-19.7 |
|  |  |  |  | obese | 69.6-126.3 | 31.1-45.3 |
|  |  |  | female | healthy | 45.5-49.25 | 19.19-19.85 |
|  |  |  |  | obese | 70.4-117.4 | 31.9-45.3 |
| Adolescents |  | 15-18 | male | healthy | 56-59.4 | 20.08-20.85 |
|  |  |  |  | obese | 96.8-139.9 | 34.7-49.1 |
|  |  |  | female | healthy | 53-54.4 | 20.45-20.88 |
|  |  |  |  | obese | 92-129.5 | 35.5-49.7 |

**Table S2. Organ volume scaling factors for virtual children and adolescent with obesity (Gerhart et al., 2022).**

| **Organ** | **Scaling Factor** |
| --- | --- |
| Bone | 6% |
| Brain | 4% |
| Gonads | 14% |
| Heart | --- |
| Kidneys | 15% |
| Large Intestine | 14% |
| Liver | 15% |
| Lungs | 14% |
| Muscle | 15% |
| Pancreas | 14% |
| Small Intestine | 14% |
| Spleen | 25% |
| Stomach | 14% |

No significant increase in size with obesity reported for heart.

**Table S3. Predicted pharmacokinetic parameters of tirzepatide after different single subcutaneous dose administration to pediatric population.**

| **Parameters** | **Dose** | **Weight** | **AUC_0-inf_**  **[ng*h/ml]** | **C_max_**  **[ng/ml]** | **T_1/2_**  **[h]** | **T_max_**  **[h]** |
| --- | --- | --- | --- | --- | --- | --- |
| Children | 5mg | normal | 188705.71 | 759.53 | 163.25 | 40.25 |
|  |  | obese | 178183.58 | 545.85 | 261.13 | 46.00 |
|  | 4.375mg | normal | 164942.52 | 663.89 | 163.24 | 40.25 |
|  |  | obese | 155745.65 | 477.11 | 261.18 | 46.00 |
|  | 3.75mg | normal | 141645.14 | 570.12 | 163.23 | 40.25 |
|  |  | obese | 133747.79 | 409.72 | 261.14 | 46.00 |
|  | 3.125mg | normal | 117881.88 | 474.47 | 163.22 | 40.25 |
|  |  | obese | 113309.80 | 340.99 | 261.14 | 46.00 |
|  | 2.5mg | normal | 94585.68 | 380.70 | 163.24 | 40.25 |
|  |  | obese | 89311.83 | 273.60 | 261.17 | 64.00 |
| Early adolescents | 5mg | normal | 138704.01 | 611.05 | 152.09 | 39.00 |
|  |  | obese | 130886.00 | 440.61 | 227.93 | 44.25 |
|  | 4.375mg | normal | 121237.85 | 534.10 | 152.10 | 39.00 |
|  |  | obese | 114404.00 | 385.12 | 227.97 | 44.25 |
|  | 3.75mg | normal | 104113.92 | 458.66 | 152.10 | 39.00 |
|  |  | obese | 98245.26 | 330.73 | 227.95 | 44.25 |
|  | 3.125mg | normal | 86647.50 | 381.71 | 152.10 | 39.00 |
|  |  | obese | 81763.31 | 275.24 | 227.94 | 44.25 |
|  | 2.5mg | normal | 69523.41 | 306.28 | 152.10 | 39.00 |
|  |  | obese | 65604.49 | 220.85 | 227.94 | 44.25 |
| Adolescents | 5mg | normal | 116082.35 | 541.39 | 142.35 | 38.00 |
|  |  | obese | 107070.60 | 354.25 | 224.10 | 44.75 |
|  | 4.375mg | normal | 101464.44 | 473.21 | 142.34 | 38.00 |
|  |  | obese | 93587.58 | 309.64 | 224.10 | 44.75 |
|  | 3.75mg | normal | 87133.35 | 406.37 | 142.35 | 38.00 |
|  |  | obese | 80369.03 | 265.90 | 224.07 | 44.75 |
|  | 3.125mg | normal | 72515.48 | 338.20 | 142.34 | 38.00 |
|  |  | obese | 66886.04 | 221.29 | 224.08 | 44.75 |
|  | 2.5mg | normal | 58184.48 | 271.36 | 142.35 | 38.00 |
|  |  | obese | 53667.43 | 117.56 | 224.07 | 44.75 |

AUC_0-inf_, area under the curve from 0 to infinity time; C_max_, the maximum concentration; T_1/2_ half-life; T_max_, the time to reach peak concentration.

**
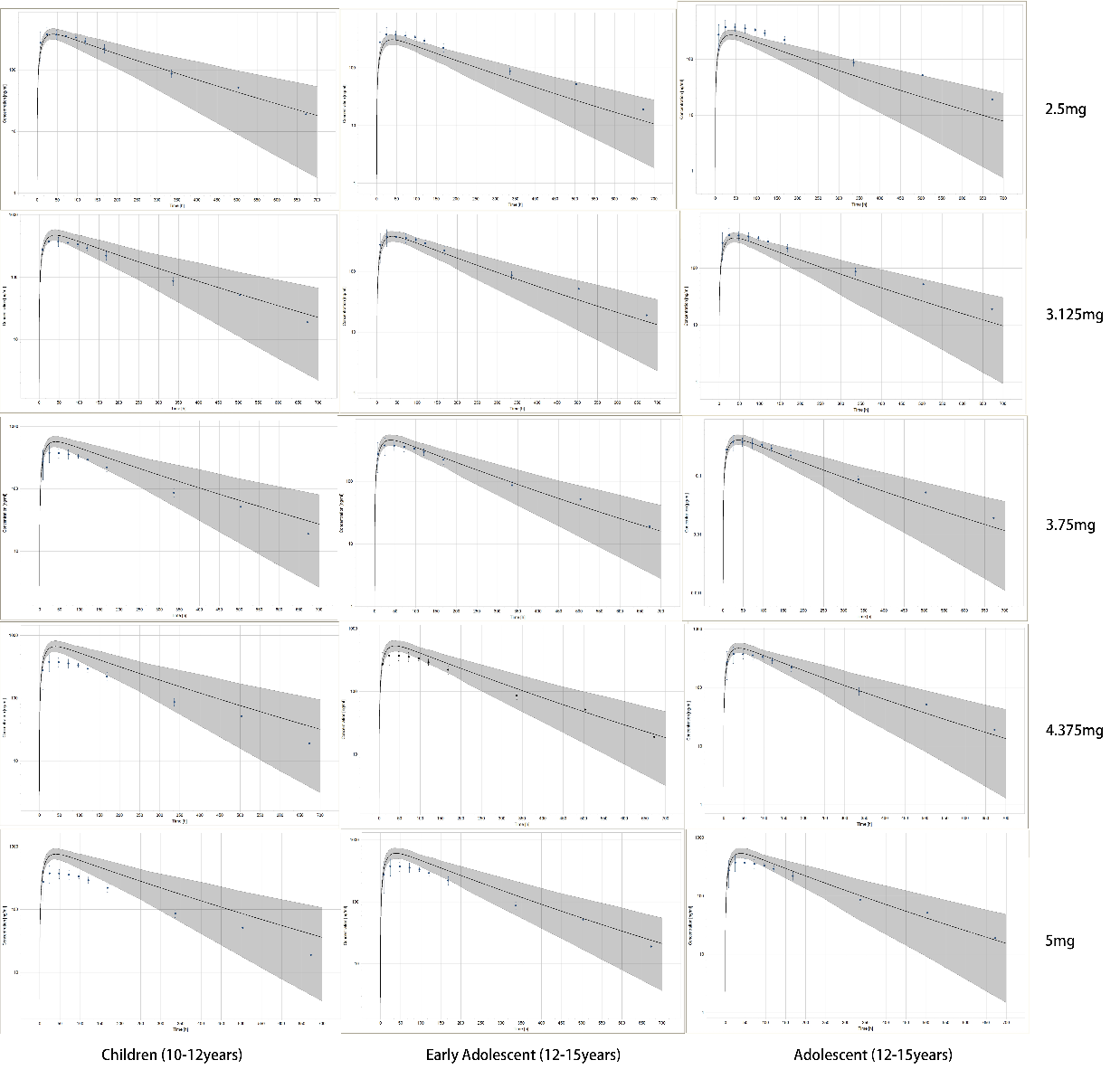
Figure S1. Predicted concentration-time profiles at simulated doses in healthy children and adolescent population. Observed data in adults at 5mg single dose is shown as black circles ± standard deviation if available.**

**
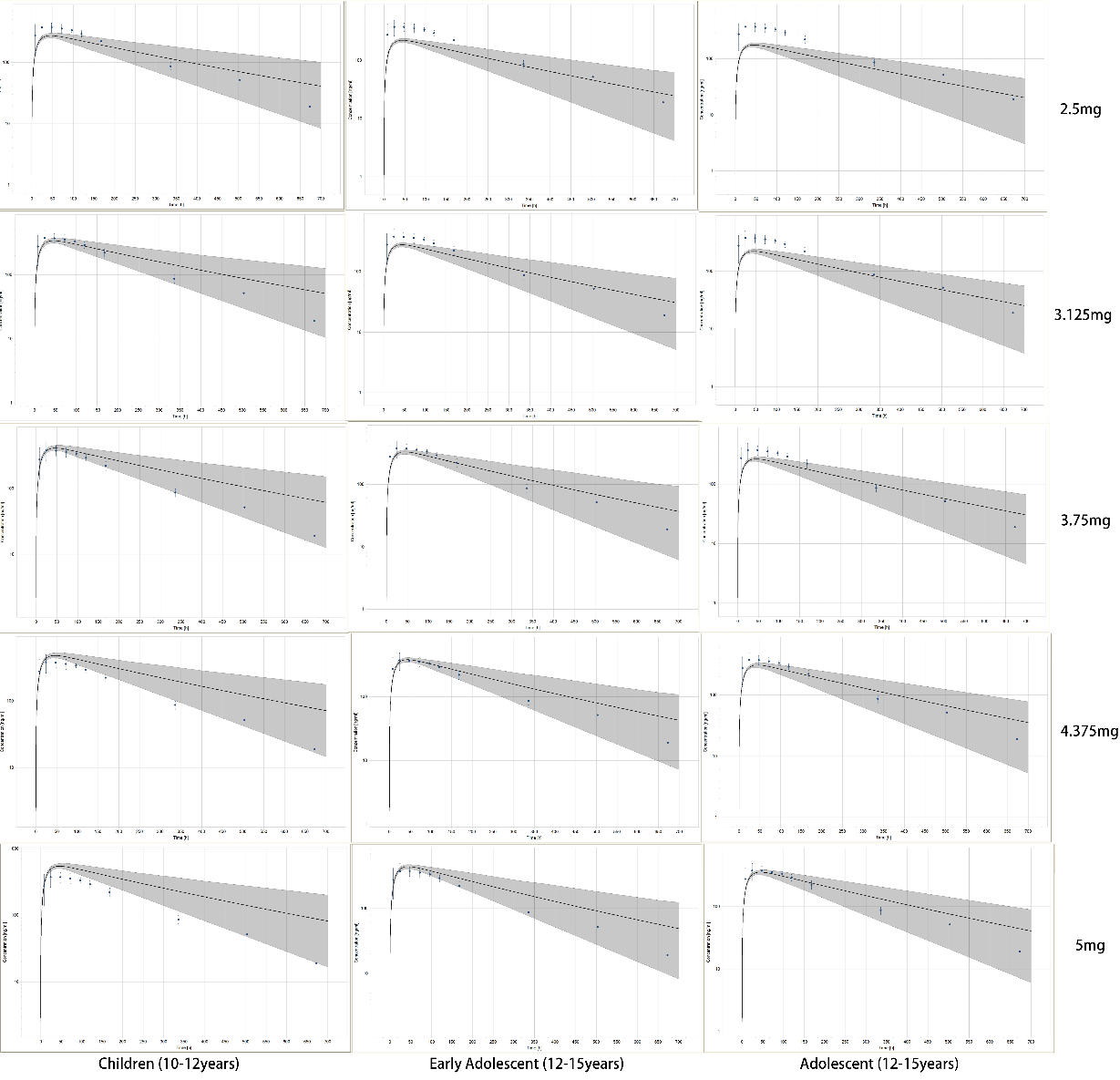
Figure S2. Predicted concentration-time profiles at simulated doses in obese children and adolescent population. Observed data in adults at 5mg single dose is shown as black circles ± standard deviation if available.**

Reference:

Gerhart, J.G., Carreño, F.O., Edginton, A.N., Sinha, J., Perrin, E.M., Kumar, K.R., et al. (2022). Development and Evaluation of a Virtual Population of Children with Obesity for Physiologically Based Pharmacokinetic Modeling. *Clin Pharmacokinet* 61(2)**,** 307-320. doi: 10.1007/s40262-021-01072-4.
